# Cardiotoxicity Associated with Chemotherapy Used in Gastrointestinal Tumours

**DOI:** 10.3390/medicina57080806

**Published:** 2021-08-06

**Authors:** Liliana Maria Radulescu, Dan Radulescu, Tudor-Eliade Ciuleanu, Dana Crisan, Elena Buzdugan, Dragos-Mihai Romitan, Anca Dana Buzoianu

**Affiliations:** 1 Department of Pharmacology, Toxicology and Clinical Pharmacology, Iuliu Hațieganu University of Medicine and Pharmacy, 400005 Cluj-Napoca, Romania; lili_m_radulescu@yahoo.com (L.M.R.); abuzoianu@umfcluj.ro (A.D.B.); 2 Department of Cardiology, Cluj Municipal Hospital, 400005 Cluj-Napoca, Romania; buzelena@yahoo.com (E.B.); romitan.mihai@yahoo.com (D.-M.R.); 3 Department of Internal Medicine, Iuliu Hațieganu University of Medicine and Pharmacy, 400005 Cluj-Napoca, Romania; crisan.dc@gmail.com; 4 Department of Oncology, Iuliu Hatieganu University of Medicine and Pharmacy, 400015 Cluj-Napoca, Romania; tudor.ciuleanu@yahoo.com; 5 Department of Internal Medicine, Cluj Municipal Hospital, 400139 Cluj-Napoca, Romania

**Keywords:** gastrointestinal cancer, cardiotoxicity, echocardiography, cardiac magnetic resonance imaging

## Abstract

Cardiotoxicity is a well-recognised side effect of cancer-related therapies with a great impact on outcomes and quality of life in the cancer survivor population. The pathogenesis of chemotherapy-induced cardiotoxicity in patients with gastrointestinal cancers involves various molecular mechanisms, and the combined use of various chemotherapies augments the risk of each drug used alone. In terms of cardiotoxicity diagnosis, novel biomarkers, such as troponins, brain natriuretic peptide (BNP), myeloperoxidases and miRNAs have been recently assessed. Echocardiography is a noninvasive imaging method of choice for the primary assessment of chemotherapy-treated patients to generally evaluate the cardiovascular impact of these drugs. Novel echocardiography techniques, like three-dimensional and stress echocardiography, will improve diagnosis efficacy. Cardiac magnetic resonance (CMR) can evaluate cardiac morphology, function and wall structure. Corroborated data have shown the importance of CMR in the early evaluation of patients with gastrointestinal cancers, treated with anticancer drugs, but further studies are required to improve risk stratification in these patients. In this article, we review some important aspects concerning the cardiotoxicity of antineoplastic drugs used in gastrointestinal cancers. We also discuss the mechanism of cardiotoxicity, the role of biomarkers and the imaging methods used in its detection.

## 1. Introduction

Gastrointestinal (GI) cancer represents an important cause of cancer-related deaths being a major public health problem that encompasses cancers of the oesophagus, stomach, colon and rectum [1]. Colorectal cancer is the most commonly diagnosed GI cancer and comprises 9% of the global cancer burden, while the other GI cancers are not negligible at all [1,2].

As overall cancer-related deaths decline, chemotherapy-associated cardiotoxicity increases and has a great impact on the outcome and life-quality of cancer survivor patients. They have an important cardiovascular (CV) risk [3], and should be considered, as recommended by the ACC/AHA guidelines, as a stage A heart failure (HF) [4]. Lenneman et al. showed a rise in the number of heart transplants in patients with cardiomyopathy, due to anthracycline therapy [5]. Others have shown that the greatest risk is in those treated with a high dose of anthracyclines or when radiotherapy is associated. Moreover, patients treated with low-dose anthracyclines or trastuzumab alone, are also at high risk, especially if they are older (>60 years), have cardiac dysfunction, previous myocardial infarction, valvular heart disease, or multiple CV risk factors [6]. Therefore, active CV screening is strongly advised, especially in those who are at a high risk to develop CV disease [7,8].

## 2. Mechanism of Cardiotoxicity

Chemotherapy-induced cardiotoxicity is defined as a reduction in left ventricle (LV) ejection fraction (LVEF) below 50%, a reduction from baseline with >10% or a Global Longitudinal Strain (GLS) reduction of >15% from baseline [7]. It manifests as cardiac dysfunction, valve or pericardial disease, arrhythmias, pulmonary hypertension, or other vascular abnormalities. Doxorubicin is most frequently accounted for HF [9], while ibrutinib for arrhythmias [10], 5-fluorouracil (5-FU) for coronary spasm, vascular endothelial growth factor (VEGF) inhibitors for arterial hypertension, and paclitaxel for conduction abnormalities.

Cardiotoxicity was initially divided in two categories based on its reversibility, but this classification is no longer available, due to several mismatches [11]. Moreover, coronary artery disease may occur after treatment with tyrosine kinase inhibitors (TKIs), drugs that may increase the risk of intrastent thrombosis [12,13]. VEGF inhibitors prevent angiogenesis and were associated with decreased nitric oxide production, vasoconstriction and hypertension [12,14], thus potentiating endothelial dysfunction, atherosclerosis and thrombotic microangiopathy [15,16]. Other chemotherapeutics can cause atrial fibrillation, QT prolongation, ventricular arrhythmias and sudden cardiac death [17,18].

### 2.1. Anthracyclines

Anthracyclines may produce cardiotoxicity by interacting with both topoisomerase II (topoII) isoforms, topoIIα and topoII [19], and by enhancing cellular apoptosis and reactive oxidant species (ROS) synthesis. Genetic analysis identified genetic loci that are involved in cell uptake and carbonyl redox cycling, which also transport anthracyclines within the cells [20]. Concomitant drugs which are also transported by these proteins, such as digoxin, diltiazem, or verapamil, may increase the cytoplasmatic concentration of anthracyclines [21]. The most common compounds used in GI cancers are doxorubicin, daunorubicine, epirubicin and idarubicin.

Cardiac histopathological changes associated with anthracyclines are mainly represented by cytoplasmatic vacuolisation, myofibrillar and myocardial sarcomere disarray [22,23].

Late-onset cardiotoxicity is the most frequently encountered. Cardinale et al. have shown that 98% of cardiotoxicity cases occurred within the first year after treatment [24].

### 2.2. Fluoropyrimidines

Fluoropyrimidines may be cardiotoxic [25], even in patients without CV diseases [26]. Fluoropyrimidines (5-FU, capecitabine) is frequently used in solid tumours, as it is the standard treatment in colorectal cancer, and increase the radiosensitivity of tumours [27]. 5-FU acts as an S-phase antimetabolite, interfering with DNA synthesis, repair and elongation [28].

Being metabolised to 5-FU, capecitabine has similar cardiotoxicity as 5-FU [29]. Coronary spasm is another mechanism of fluoropyrimidines’ cardiotoxicity, which was suggested not to be endothelial-dependent [30,31]. Moreover, 5-FU increased the levels of endothelin-1 and von Willebrand factor [32]. 5-FU cardiotoxicity may also be secondary to enhanced synthesis of fluoroacetate and fluorocitrate, cellular apoptosis and ROS synthesis [25].

Therefore, fluoropyrimidines may cause chest pain, Takotsubo cardiomyopathy, Kounis syndrome, arrhythmias and cardiac arrest. The incidence of cardiac events in high-dose 5 FU treated patients may be as high as 7.6% [25,33,34].

### 2.3. Platinum-Based Compounds

Platinum-based compounds are important chemotherapies in gastrointestinal tumours, which is used in oesophageal cancer, colorectal cancer (FOLFOX). Cisplatin is another commonly used drug that is associated with significant toxicity [35], while carboplatin has fewer side effects. They interact with DNA and determine ROS production [36]. Additionally, cisplatin has been associated with cardiomyocyte apoptosis secondary to activating the transmembrane protein kinase RNA-like ER kinase pathway [37].

The incidence of thromboembolic side effects was as high as 10 to 20% in different studies. Endothelial dysfunction can also play a role in cisplatin-associated cardiotoxicity, which is associated with increased levels of von Willebrand factor [38]. Studies have shown an increased incidence of arterial hypertension and LV hypertrophy in patients treated with cisplatin, in a cumulative dose-dependent manner [39].

### 2.4. Human Epidermal Growth Factor Receptor 2 (HER2) Antagonists

Trastuzumab, a HER2 antagonist, has recently been proven beneficial in some HER2 positive gastric and colorectal cancers [40,41]. Trastuzumab cardiotoxicity was first described in breast cancer trials, especially when combined therapies were used [42]. Pertuzumab, another HER2 antagonist, used in gastric cancer, proved similar cardiotoxicity as trastuzumab, but without the same additive toxicity [43].

Neuregulin, an ErbB receptor found in the CV system, may be involved in HER2 therapy-induced cardiotoxicity. Neuregulin, ErbB2 and ErbB4 receptor tyrosine kinases have been documented to play important roles in cellular cross-communication and stress-response [44]. The disruption of ErbB2 expression may cause dilated cardiomyopathy [45], whereas the suppression of neuregulin/ErbB signal may prevent recovery from ischemic injury [46]. Moreover, anti-ErbB2 therapy may induce ROS and cell apoptosis, and as a consequence, increased levels of troponins I and cardiac myosin light chain-1 [47]. Recently, it has been documented that trastuzumab may provoke endothelial and mitochondrial dysfunction [48].

### 2.5. Immune Checkpoint Inhibitors (ICI)

ICI are a class of drugs that block the immune down-regulators, such as programmed cell death-1 (PD1), PD ligand-1 (PDL1), cytotoxic T-lymphocyte antigen 4 (CTLA-4) [49]. Nivolumab and pembrolizumab are anti-PD1 agents used in colorectal and gastric cancers. In murine models, CTLA-4 and PD1 exert cardioprotective effects. In turn, the blockage of CTLA-4 was associated with fatal myocarditis [50]. Some pericardial antigens may be activated by ICI, leading to pericarditis, however this is a less frequent side effect [51].

According to recent data, ICI-related myocarditis may occur in 1.14% of patients when the drugs are used alone and in 2.4% of patients if the drugs are used in combination therapy. Nonetheless, the risk of developing myocarditis is shown to be lower with anti-PD1 drugs, when compared to anti-PDL1 and anti-CTLA4 drugs [52]. Studies have shown that it usually appears after a median follow-up period of 2 to 3 months, but it can occur anytime during treatment, having a high mortality risk [52,53,54]. Additionally, Takotsubo syndrome related to ICI had been reported [55].

### 2.6. Angiogenesis Inhibitors

VEGFs are involved in angiogenesis, endothelial cell survival, vasomotricity and cardiac contractile function, and their inhibitors have been associated with HF and arterial hypertension [56]. Bevacizumab, an antibody-based anti-VEGF, was also associated with atherothrombotic events [57]. Moreover, some studies reported preeclampsia–like syndrome secondary to treatment with angiogenesis inhibitors [58]. Additionally, these compounds were associated with irreversible diastolic dysfunction [59].

It has been shown that these drugs can increase the risk of both ischemic and haemorrhagic stroke, especially when bevacizumab is used. This risk is also higher in patients with colorectal cancers, and it doubles when a double dose is used [60]. HF was encountered more frequently in those treated with TKIs, and it is caused by the tyrosine kinase, AMP-activated kinase and platelet-derived growth factor receptors inhibition [58,61].

VEGFs inhibitors associated cardiotoxicity is usually divided into two types: (1) On-target toxicity, which is due to the inhibition of CV-expressed kinases and it occurs mostly with imatinib treatment; and (2) off-target toxicity, encountered with sunitinib therapy, in which CV side effects are due to inhibition of non-targeted kinases [62].

## 3. Diagnosis Methods of Chemotherapy-Induced Cardiotoxicity in GI Cancers

The evaluation of cardiotoxicity has an important role in patients treated with cancer therapies. Detecting subclinical changes in cardiac function represents the subject of interest of many studies. Echocardiography and cardiac magnetic resonance imaging (CMR) is the most used techniques. Cardiac biomarkers are used to increase the sensitivity of imaging methods in detecting cardiotoxicity, mainly in the early stages [7,63].

### 3.1. Biomarkers

Biomarkers are considered to be a cost-effective alternative to other diagnostic techniques for screening subclinical changes in cardiac function. The most useful serum biomarkers used for the assessment of chemotherapy-based cardiotoxicity are represented by cardiac troponins (cTn) I/T, brain natriuretic peptide (BNP), C-reactive protein, myeloperoxidase (MPO), growth differentiation factor 15 (GDF-15), galectin-3, soluble interleukin 1 receptor-like 1 (ST2) and some microRNAs (miRNA). These can be used in the risk stratification and follow-up of cardiotoxicity and in evaluating cardio-protection. Current guidelines recommend using such biomarkers in patients treated with chemotherapy, by determining their value at baseline, during chemotherapy and after chemotherapy [7,64].

#### 3.1.1. Troponins

Cardiac troponins I and T are organ-specific biomarkers used to diagnose myocardial injury, which are also reliable in chemotherapy-based cardiotoxicity. In a recently published meta-analysis, the significant link between cardiac troponins and chemotherapy was proved. Studies have found that cTn was able to detect early cardiac injury more accurately than natriuretic peptides [7]. Increased serum levels of cTn were frequently reported in patients treated with anthracyclines, even within the first 72 h of anthracycline treatment and at one month after therapy cessation [65]. Additionally, Jones et al. showed these biomarkers to be significantly increased after the fourth, the fifth and even after the sixth cycle of chemotherapy [66]. Garrone et al. found that high levels of cTn were positively correlated with the risk of developing HF [67], while other studies demonstrated that these biomarkers were predictors of early cardiac dysfunction, having important roles in detecting cardiotoxicity [68,69,70].

Furthermore, elevations in serum cTn were observed with trastuzumab treatment and were associated with cardiac dysfunction [70]. cTn has been shown to play important roles even in the prediction of LV dysfunction [68,71]. Furthermore, cTn may identify subclinical forms of ICI-based myocarditis, which are associated with a worse prognosis [52].

#### 3.1.2. BNP

BNP, another serum biomarker, having an important role to diagnose chemotherapy-induced cardiotoxicity, is associated with increased LV filling pressures [72]. In patients with impaired LV function secondary to anthracycline therapy, Feola and al. documented significantly higher levels of BNP at baseline and at follow-up, in comparison with patients without LV dysfunction. Patients who develop cardiac side effects, due to chemotherapy, have higher BNP levels at baseline [73]. This biomarker has an important predictive value in documenting the development of chemotherapy-induced HF [74]. NT-proBNP levels are directly correlated with anthracycline treatment and are also associated with an increased 1-year mortality, as shown by Iuliis et al. [75].

#### 3.1.3. Other Biomarkers of Use in Detecting Cardiotoxicity

MPO and GDF-15 are associated with impaired LV function and even with decreased LVEF [76]. miRNAs are also studied in patients treated with both anthracyclines and trastuzumab, and some studies have shown that several miRNAs are increased in the first 24 h after chemotherapy, but further studies are needed [77].

### 3.2. Echocardiography

Echocardiography is the main imaging method used in diagnosing cardiotoxicity, which comprehensively evaluates heart morphology and function [7,78]. LVEF is the most common marker of LV systolic function, which is assessed using the modified biplane Simpson’s method. Although, it has low sensitivity and high temporal variability [79,80], these shortcomings may be improved using contrast echocardiography [81]. Three-dimensional echocardiography (3DE) has increased accuracy in evaluating heart function, and the lowest variability [82]. Other echocardiography parameters, such as myocardial performance index (MPI), which can identify subclinical cardiotoxicity in patients treated with 5-FU and anthracyclines, and mitral annular plane systolic excursion (MAPSE), may also be used [83,84]. LVEF <55% was associated with a significantly increased risk to develop HF, due to HER2 antagonists and anthracyclines [85,86].

Diastolic dysfunction was the first to occur in most of the chemotherapy-treated patients. In some studies, the E/A ratio was impaired, due to doxorubicin, along with changes in isovolumetric relaxation time (IVRT), even after the first dose [87,88,89]. IVRT was also able to predict LVEF changes [87].

3DE has comparable accuracy with CMR and multi-gated acquisition scan (MUGA) in terms of LVEF evaluation [90,91]. The most sensitive 3DE markers of early cardiotoxicity, are represented by an LVEF <55%, and end-systolic volume index >29 mL/m^2^, and a global longitudinal strain (GLS) <−17.5%, having comparable efficacy with CMR [92]. LV strain is a novel technique used for monitoring cardiotoxicity, and a reduction in GLS of >15% is considered a marker of cardiotoxicity [64]. A 6-months decrease in GLS >15% may identify cardiac dysfunction in breast cancer patients at 12 months after stopping chemotherapy [70].

3DE speckle-tracking imaging (STI) overcomes the limitations of 2DE STI in terms of identifying early cardiac changes [93]. Global circumferential strain (GCS), and global radial strain are decreased early after anthracycline treatment, despite preserved LVEF. Global area strain curve (GAS) was also associated with subclinical LV changes [94]. In patients treated with trastuzumab, GLS significantly changed at 6-months after chemotherapy [95]. GLS was strongly associated with cardiac events among patients with ICI-related myocarditis [96].

During anthracycline therapy, changes in LV torsion, twisting and untwisting occurred earlier than an LVEF drop. Apical torsion, twisting and untwisting rates after chemotherapy, were correlated with prolonged IVRT, but didn’t predict LVEF decrease [97].

Echocardiography is used in detecting wall motion abnormalities, due to ischemia or evaluation of LV wall hypertrophy secondary to arterial hypertension in patients treated with fluoropyrimidines, platinum-based chemotherapy and anti-VEGFs. Stress echocardiography (SE) may be used to reveal subclinical changes in LV function [98]. In patients treated with doxorubicin, the sensitivity of resting LVEF may increase from 50% to 90% when SE is used [99]. SE can be used to assess stable coronary artery disease [100], and in those treated with 5-FU and bevacizumab. It can also evaluate LV’s contractile reserve, a 5-units decrease having a predictive value for future LVEF decline [101].

### 3.3. Cardiac Magnetic Resonance Imaging

CMR is used in evaluating cardiac structure and function, and it is the gold standard for accurate assessment of ventricles’ functions and volumes. It may detect myocardial oedema and fibrosis, and early changes in cardiac function [102]. Mild LV dysfunction occurs early during treatment with anthracyclines and trastuzumab with a 3% decrease in LVEF at one month and 5% at four months after stopping chemotherapy. Nearly all chemotherapy-treated patients developed RV dysfunction, an impairment that persisted during follow-up at twelve months, even though in some patients, LV function recovered at four months [103].

Myocardial oedema, inflammation and interstitial fibrosis, occur early before LV dysfunction and LVEF impairment [104]. Myocardial oedema can be detected by CMR, mostly on T2-weighted signal intensity [105]. Myocardial oedema and fibrosis are associated with higher mortality [106]. Steady-state free-precession (SSFP) CMR provides data on wall motion segmental and global changes, and may detect subtle changes [107]. Cine-CMR detects subtle LV morpho-functional abnormalities, even in patients treated with low dose anthracyclines. LV mass also changes during cancer therapy, mostly in anthracycline-treated patients [108]. Interestingly, a smaller LV mass correlated with the cumulative dose of anthracycline, a negative prognostic factor [90,109]. An LV mass less than 57 g/m^2^ was associated with a higher risk of HF and CV deaths [110]. RV dysfunction was also observed during anthracycline treatment or anthracycline-trastuzumab combination, and it was correlated with higher mortality [111,112].

Late-gadolinium enhancement (LGE) is used in evaluating myocardial fibrosis [113,114], and data from studies are contradictory [115]. LGE is infrequently observed in patients treated with trastuzumab and anthracyclines, and its absence doesn’t exclude myocardial fibrosis [116,117].

T1 mapping is used to quantify extracellular volume fraction (ECV), which indicates interstitial fibrosis [63]. On T1 weighted sequences, abnormal gadolinium accumulation is observed in cancer survivors [118], and may detect subclinical changes. Furthermore, ECV was significantly associated with histologically proven myocardial interstitial fibrosis [119,120]. Patients treated with anthracycline had higher ECV and associated diastolic dysfunction [116]. CMR can determine intracellular water lifetime using T1 mapping in order to properly characterise the myocardial tissue [121]. Anthracycline therapy determines a significant decrease of the LVEF, LV mass and intracellular water lifetime [119].

Early gadolinium enhancement (EGE) detects myocardial injury in patients with myocarditis [122], is included in the Lake Louise criteria. After anthracycline treatment, EGE significantly predicted a decline in LVEF [118]. CMR can also properly evaluate patients with ICI-related myocarditis and preserved LVEF, especially in combination with using LGE [123,124].

Furthermore, the myocardial consequences of arterial hypertension secondary to TKIs can be evaluated by CMR [64]. Vascular stiffness may be assessed by CMR, by aortic phase-contrast imaging. Pulse wave velocity (PWV) increases after anthracycline administration, which is associated with LV dysfunction and CV adverse effects, but its impact on the outcome of cancer patients needs further studies [108]. Lastly, T2 relaxation time is another marker of early LV dysfunction, which occurs sooner than ECV [125], but the studies are just at the beginning.

## 4. Risk Stratification and Surveillance during and after Chemotherapy

**Figure 1:**
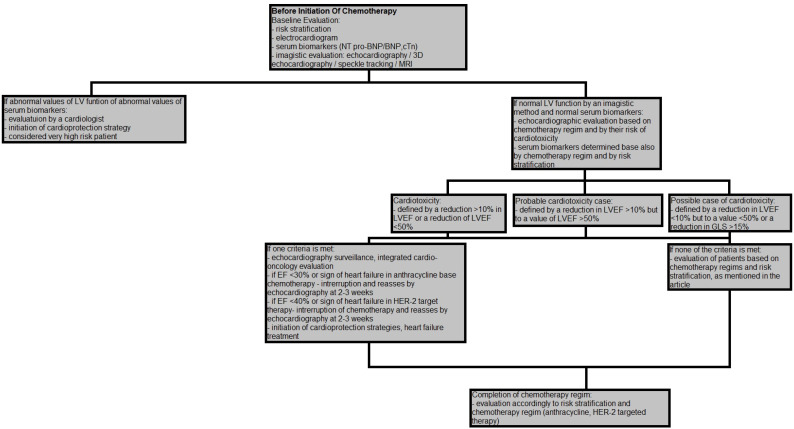
Standard of care and a surveillance plan.

An important aspect in evaluating patients treated with chemotherapy is establishing their baseline cardiovascular risk and their risk of developing cardiotoxicity [126]. This approach will allow for a universal standard of care and a surveillance plan [63,64,78] (Figure 1). According to their risk level (low, medium, high, very high), we could establish the risk of developing cardiotoxicity, which is <2%, 2–9%, 10–19%, ≥20%, respectively [7].

According to the recently published position paper by the European Society of Cardiology (ESC), risk factors that should be considered are represented by previous cardiovascular diseases, co-existing medical disease, demographic traits and lifestyle factors that are accounted as cardiovascular risk factors, elevated serum biomarkers before initiation of chemotherapy and previous chemotherapy. High risk patients are considered those with one or more high risk factors or patients with medium risk factors with a sum of >5 points. Very high risk patients are considered those with one or more very high risk factors [7].

Very high risk factors are represented by pre-existing heart failure or cardiomyopathy in patients treated with anthracycline, HER-2 targeted therapies and VEGF inhibitors.

High risk factors are represented by ischemic cardiomyopathy (myocardial infarction, coronary artery by-pass grapht, stable angina), severe valvular heart disease, low LVEF at baseline (<50%), age >80 years, previous chemotherapy (anthracycline or HER-2 therapies) and radiotherapy to the left side of the chest or mediastinum [7].

Serum biomarkers are used in surveillance during and after chemotherapy, and according to ESC position paper in high risk CV patients treated with anthracycline BNP/NT-proBNP, cTn should be measured at baseline, before cycles 2, 4 and 6 or before every cycle and at 3 and 6 months following chemotherapy or at 12 months after the final cycle. In low and medium CV risk patients, these biomarkers should be determined at baseline, before the fifth cycle and at 12 months after chemotherapy. In patients treated with HER-2 targeted therapies for gastric cancer, serum biomarkers should be measured at baseline, before every cycle for 3–6 months and then every 3 months for the remaining treatment in the first year. Following chemotherapy serum biomarkers are measured only if the patient is symptomatic [126].

According to the British Society of Echocardiography and British Society of Cardio-Oncology guidelines published recently regarding the frequency of echocardiography during chemotherapy, high risk patients treated with anthracycline, should be evaluated every two cycles or every cycle if the dose of doxorubicin is >240 mg/m^2^. In patients treated with HER-2 targeted therapy, echocardiography should be done every two or three cycles for three months, then to every fourth cycle in the first year [78].

## 5. Future Directions

There are still many uncertainties in evaluating patients undergoing cancer therapy, especially in detecting subclinical changes. Some new imaging techniques have been investigated to detect early cardiotoxicity, such as 3DE STI and new CMR techniques, which proved to have promising results. With the development of these new imaging techniques, there is a need for more clinical trials, for better cancer treated patients’ surveillance, to improve their outcome. Resorting to cardiac imaging methods and cardiac biomarkers may help to detect subclinical changes in cardiac function, while the damage is still reversible and cardio-protection methods are effective.

## Data Availability

Not applicable.
